# Suppressive Effects of GSS on Lipopolysaccharide-Induced Endothelial Cell Injury and ALI via TNF-*α* and IL-6

**DOI:** 10.1155/2019/4251394

**Published:** 2019-12-30

**Authors:** Lei Yi, Zengding Zhou, Yijuan Zheng, Mengling Chang, Xiaoqin Huang, Feng Guo, Quanming Zhao, Jingning Huan

**Affiliations:** ^1^Department of Burn and Plastic Surgery, Ruijin Hospital, School of Medicine, Shanghai Jiao Tong University, Shanghai, China; ^2^Department of Critical Care Medicine, Zhongshan Hospital, Fudan University, Shanghai, China; ^3^Department of Plastic Surgery, Shanghai Jiaotong University Affiliated Sixth People's Hospital, Shanghai, China; ^4^Department of Orthopedic Surgery, The Second Affiliated Hospital of Nantong University, Nantong, Jiangsu Province, China

## Abstract

*Background*. Under septic conditions, LPS induced lung vascular endothelial cell (EC) injury, and the release of inflammatory mediator launches and aggravates acute lung injury (ALI). There are no effective therapeutic options for ALI. Genistein-3′-sodium sulfonate (GSS) is a derivative of native soy isoflavone, which exhibits neuroprotective effects via its antiapoptosis property. However, whether GSS protect against sepsis-induced EC injury and release of inflammatory mediators has not been determined. In this study, we found that GSS not only downregulated the levels of TNF-*α* and IL-6 in the lung and serum of mice *in vivo* but also inhibited the expression and secretion of TNF-*α* and IL-6 in ECs. Importantly, we also found that GSS blocked LPS-induced TNF-*α* and IL-6 expression in ECs via the Myd88/NF-*κ*B signaling pathway. Taken together, our results demonstrated that GSS might be a promising candidate for sepsis-induced ALI via its regulating effects on inflammatory response in lung ECs.

## 1. Background

Genistein is the major isoflavone in soybeans and a natural tyrosine kinase inhibitor [1]. Recently, scientific interest in genistein has grown enormously owing to genistein putative multiple pharmacological properties such as antitumor, anti-inflammatory, and antioxidant effects [2, 3]. Though genistein has many beneficial effects, its poor water solubility and fat solubility limits its widespread application [4]. To improve the bioavailability and activity of genistein, Li et al. synthesized genistein-3′-sodium sulfonate (GSS), a new compound derived from genistein that protected cortical neurons from ischemia-induced neuronal apoptosis [5]. However, there is no report about the effects of GSS on bacterial infection-induced acute lung injury (ALI) under sepsis condition until now.

Sepsis is characterized by an imbalance of proinflammatory responses that induce the dysfunction of multiple organs, such as ALI and acute respiratory distress syndrome (ARDS) [6]. ALI is a severe clinical syndrome characterized by diffuse pulmonary edema. Much of the pathogenesis of ALI is due to lung vascular endothelial cell injury, which leads to increased lung vascular permeability and extensive inflammatory pulmonary infiltrates [7]. Endothelial cell injury-induced disruption of lung endothelial barrier integrity and excessive inflammatory response are central to the pathogenesis of sepsis-induced ALI [8–10]. Some studies have demonstrated that the inflammation response of lung vascular ECs in WT mice was upregulated in LPS-induced ALI [11], and blocking pulmonary inflammation effectively attenuated histopathologic lung injury [12, 13].

Sepsis can be initiated by extensive vascular endothelial cell (EC) dysfunction in response to gram-negative bacterial infection [14]. Lipopolysaccharide (LPS) is the major component of the outer surface of gram-negative bacteria and is recognized as the vital factor involved in the pathogenesis of EC injury [14]. LPS binds and activates Toll-like receptor 4 (TLR4) of ECs, which results in massive release of various proinflammatory mediators, such as interleukin 6 (IL-6) and tumor necrosis factor *α* (TNF-*α*). Overexpression of TNF-*α* and IL-6 is associated with ALI, multiple organ dysfunction syndrome (MODS), and mortality [15, 16].

Nuclear factor *κ*B (NF-*κ*B) is a ubiquitous nuclear transcription factor which plays an important role in the inflammatory response to infection and is involved in the regulation of a variety of genes activated upon inflammation including the promoter region of the TNF-*α* and IL-6 gene [17]. Our previous studies suggested that after the interaction of LPS and TLR4 in the membrane of ECs, LPS induced activation of downstream NF-*κ*B in ECs via both the Myd88-dependent manner (Myd88 pathway) and the Myd88-independent manner (GEF-H1 pathway) [18]. Recently, Jeong et al. showed that genistein had anti-inflammatory effects in LPS-stimulated microglial cells via suppression of the TLR4 signaling [19]. However, the underlying effects of GSS on LPS-induced activation of TLR4 pathway and subsequent TNF-*α* and IL-6 overexpression in lung microvascular ECs are largely unknown. These experiments were designed to investigate whether GSS confers protective effects against LPS-induced overexpression of TNF-*α* and IL-6 in lung microvascular ECs and to determine the related signaling pathway.

In the present study, we not only found that GSS protected against sepsis-induced ALI but also revealed that the above protective effects of GSS are achieved through inhibition of LPS-induced Myd88/NF-*κ*B/TNF-*α*/IL-6 signaling activation instead of GEF-H1/NF-*κ*B pathway in lung microvascular ECs. Our findings provided new perspectives on the protective role of GSS in LPS-induced lung microvascular EC injury, and the findings of this study may contribute to the prevention and treatment of sepsis-induced ALI.

## 2. Methods (We Followed the Methods of Yi et al. [20])

### 2.1. Animal Studies (ALI Model, Lung Histopathology, Ratios of Wet/Dry, Ratios of PaO_2_/FiO_2_, and Evans Blue Extravasation)

Male C57BL/6 mice (6-8 week old, 18-20 g) were obtained from the Experimental Animal Center of Ruijin Hospital, Shanghai, China. All of the mice were provided with standard laboratory food and water and housed in accordance with institutional animal care policies. All procedures and animal care were performed in accordance with the National Institutes of Health *Guide for the Care and Use of Laboratory Animals* with the approval (SYXK-2011-0113) of the Scientific Investigation Board of Shanghai Jiao Tong University School of Medicine, Shanghai, China. The animals were acclimatized to the laboratory conditions (25°C, 12 h/12 h light/dark, 50% humidity, and ad libitum access to food and water) for one week prior to experimentation. The mice were firstly pretreated with or without BAY11-7082 by intraperitoneal injection for 2 hours, and then, the sepsis-associated ALI was induced by a cecal ligation and puncture (CLP) model; the mice were anesthetized with 1% sodium pentobarbital (40 mg/kg). For histological assessment of lung injury, the mouse lungs were harvested 24 h after CLP application and were quickly removed and fixed in 10% paraformaldehyde. The paraformaldehyde-fixed lobe of the lungs was embedded in paraffin and cut into 5 *μ*m sections. H&E staining was performed using standard protocols. The slides of each group were assessed under high-power fields (sections were evaluated at ×100 magnification). For assessment of sepsis-induced lung edema, the lungs were immediately weighed to obtain the wet weight and then placed in an oven at 80°C for 48 hours to obtain the dry weight. The ratio of the wet lung to the dry lung was calculated to assess lung edema. For analysis of sepsis-induced lung vascular leak, Evans blue dye (30 ml/kg) was injected into the caudal vein 2 hours before termination of the experiment. Measurement of Evans blue accumulation in the lung tissue was performed by spectrofluorimetric analysis of lung tissue lysates as before [21]. For measurement of oxygenation index changes, the carotid arteries were cannulated, and the arterial blood samples were collected for analysis [22].

### 2.2. Cell Culture and Chemicals

For cell culture and reagents, primary mouse pulmonary microvascular endothelial cells were obtained from Angio-Proteomie (Boston, MA, US) and maintained in Angio-Proteomie Endothelial Cell Medium in a humidified 37°C, 5% CO_2_ incubator. The medium was changed at 48-hour intervals. Genistein-3′-sodium sulfonate (GSS) was obtained from Shanghai Tianxi Chemical Co., Ltd. (Shanghai, China), and BAY11-7082 was purchased from Beyotime of China. The LPS (from Escherichia coli 055:B5) was obtained from Sigma-Aldrich (St. Louis, MO). The anti-GEF-H1 rabbit monoclonal antibody (mAb), anti-Myd88 rabbit mAb, anti-NF-*κ*B p65 rabbit mAb, and anti-rabbit phospho-NF-*κ*B-p65 rabbit antibody were obtained from Cell Signaling Technologies (Danvers, MA). Alexa Fluor 594-conjugated goat anti-rabbit secondary antibody and the ProLong Gold Antifade Mountant with DAPI were obtained from Invitrogen Life Science.

### 2.3. Cell Viability Assay

The Cell Counting Kit-8 (CCK-8; CK04, DOJINDO) was used to assess cell viability according to the manufacturer's protocol. ECs were seeded in 96-well plates and cultured for 24–48 h. When the monolayer of cells was 90% confluent, the ECs were exposed to various concentrations of GSS for different times. Next, 10 *μ*l of CCK-8 solution was added to each well, and the cells were incubated for an additional 3 h. The absorbance at 450 nm was measured using a microplate reader. The cell survival rate was calculated using the average of pooled data from three separate experiments of six wells.

### 2.4. ELISA

The supernatants were assayed for TNF-*α* and IL-6 protein content using ELISA kits for TNF-*α* and IL-6 (BD Biosciences, San Diego, CA) according to the manufacturer's instructions. Briefly, each well of the 96-well plate was coated overnight with capture antibody before being washed with PBS containing 0.05% Tween; then, the supernatant was added to the appropriate wells. After having been incubated for 1 hour at room temperature, the detection antibody was added and incubated for another 1 hour. The wells were then washed with PBS/Tween, and horseradish peroxidase-conjugated streptavidin was added for further 1 hour at room temperature. Finally, the color was developed by adding peroxidase substrate to each well before reading the absorbance at 450 nm using the Dynatec plate reader (Denkendorf, Germany).

### 2.5. RT-PCR

Total RNA was prepared from ECs using TRIzol reagent (Invitrogen) according to the manufacturer's instructions. First-strand cDNA was synthesized using the oligo(dT) 15 primer and M-MLV reverse transcription technique (Promega) on 4 *μ*g of total RNA in 40 *μ*l reaction volume. Taq DNA polymerase was used to amplify the resulting cDNA with a 25 *μ*l reaction volume. The primer sequences were designed as follows: the primer sequences of TNF-*α* were 5′-ATG GCG TGG AGC TGA GAG ATA-3′ and 5′-GGG GAG GCG TTT GGG AAG GT-3′, the primer sequences of IL-6 were 5′-CAT TGC CAT TGG TCT GAG GTT C-3′ and 5′-AGT AGT CTG TAT TGC TGA TGT C-3′, and the primer sequences of GAPDH were 5′-GGT CTA CAT GGC AAC TGT GA-3′ and 5′-ACC AGG TGG TCT CCT CTG A-3′; PCR produces were seen on 2% agarose gels, following electrophoresis by ethidium bromide staining, and photographed under UV light.

### 2.6. Extraction of Nuclear and Cytosolic Fractions

The extraction and isolation of nuclear and cytoplasmic proteins were performed according to the manufacturer's instructions using a nuclear and cytoplasmic protein extraction kit (Beyotime, Jiangsu, China). Briefly, after treatment, ECs were washed with PBS and collected by centrifugation. EC pellets were resuspended in 200 ml extraction buffer A and incubated for 15 min on ice, and then, extraction buffer B was added. After centrifugation, supernatants were removed and stored at -80°C until analyzed by gel electrophoresis. Pellets, which contained the nuclei, were resuspended in 50 ml of nuclear extraction buffer, and nuclear proteins were extracted by shaking the samples. Afterwards, samples were centrifuged and the supernatants were removed and analyzed using gel electrophoresis. The validation of the method used to isolate the cytosolic and nuclear fractions (histone-H3 was used as a loading control for nuclear proteins, and GAPDH was used as the loading control for cytoplasm proteins) was checked using Western blot analysis.

### 2.7. Western Blot Assay

The total protein was extracted from EC monolayers or lung tissue extracts, and then, the target protein expression was probed with specific antibodies. The equal amounts of cells or lung tissue extracts were separated by 6–12% sodium dodecyl sulfate polyacrylamide gel electrophoresis (SDS-PAGE) according to the molecular weight of the target proteins and electrotransferred to PVDF membrane. The membranes were blocked in Tris-buffered saline (TBS) and Tween-20 containing 5% nonfat milk at room temperature for 1 hour at room temperature and then incubated with antibodies to the target proteins (GEF-H1, Myd88, NF-*κ*B p65, and P-NF-*κ*B p65 dilutions were 1 : 1000) overnight at 4°C. The membranes were incubated with the appropriate HRP-linked secondary antibodies (1 : 2000) at room temperature for 1 hour. The signal intensities were compensated by glyceraldehyde 3-phosphate dehydrogenase (GAPDH) as internal controls. Finally, the bands were developed with a Western blot luminal reagent (Millipore, Billerica, MA). Protein bands were quantified using ImageQuantR software (Molecular Dynamics, Sunnyvale, CA).

### 2.8. Transfection with Myd88 siRNA

Stealth RNA interference duplexes against Myd88 were designed and synthesized by Gene pharma Technologies (Shanghai, China). Myd88 siRNA molecules were transfected individually into ECs using Lipofectamine RNAiMAX (Carlsbad, CA) according to the manufacturer's instructions. Duplex siRNA was constructed against sequences coding for Myd88 (5′-CCG GAU GGU GGU GGU UGU CUC UGA U-3′). A scrambled, negative control siRNA (5′-UUC UCC GAA CGU GUC ACG UTT-3′) was also included. The ability of RNA interference molecules to knockdown target protein expression was analyzed by Western blot analysis.

### 2.9. Confocal Immunofluorescent Analysis

For active NF-*κ*B staining, confluent EC monolayers were washed twice with prewarmed PBS (pH 7.4), fixed in 4% paraformaldehyde for 15 minutes, and then permeabilized with PBS containing 0.1% Triton X-100 and 1% bovine serum albumin for 10 minutes. After washing in PBS, the monolayers were incubated with the anti-NF-*κ*B p65 rabbit mAb (1 : 100) overnight at 4°C. The ECs stained with active NF-*κ*B were further incubated with the Alexa Fluor 594-conjugated goat anti-rabbit secondary antibody (1 : 500) for 1.5 hours. Finally, ECs were sealed by ProLong Gold Antifade Mountant with DAPI, and then, the monolayers were imaged with confocal microscopy.

### 2.10. Statistical Analysis

The results are expressed as the means ± SEM of at least 3 independent experiments. Data are expressed as the mean and standard error. Student's *t*-tests and ANOVAs were used as appropriate. The significance was accepted at *P* < 0.05.

## 3. Results

### 3.1. GSS Inhibits Sepsis-Induced ALI and Lung Vascular Hyperpermeability

**Figure 1:**
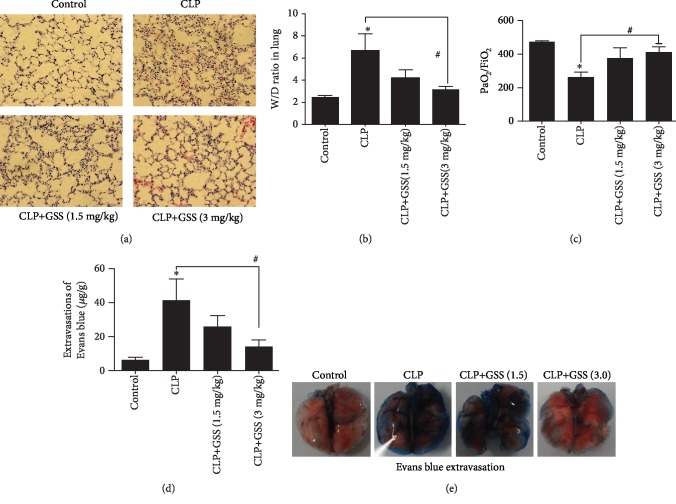
GSS inhibits sepsis-induced acute lung injury and vascular barrier dysfunction in mice. The mouse lungs harvested 24 h after CLP application. (a) Sepsis in C57BL/6J mice was induced by the CLP procedure after pretreatment of GSS, and the histological analysis of lung tissue was analyzed by hematoxylin and eosin staining. (b) Wet/dry ratio of lungs was represented as a histogram according to data. (c) The PaO_2_/FiO_2_ ratio was detected after CLP in mice with and without GSS treatment. (d) The quantitative analysis of Evans blue extravasation was performed by spectrophotometric analysis of Evans blue extracted from the lung tissue samples. (e) Evans blue dye was injected 2 hours before termination of the experiment. ^∗^*P* < 0.05 versus negative control. ^#^*P* < 0.05 versus the corresponding CLP treatment group.

In order to find out whether GSS blocks sepsis-induced lung vascular barrier disruption and ALI, the effects of GSS on sepsis-induced lung histologic changes were firstly examined in this study. We found that pretreatment with GSS obviously ameliorated sepsis-induced lung pathologic changes, mainly including abundant inflammatory cell infiltration and interstitial edema (Figure 1(a)). Moreover, effects of GSS on sepsis-induced lung vascular barrier disruption were detected by the wet/dry weight ratio changes (Figure 1(b)). In this study, we also found that GSS reverses sepsis-induced PaO_2_/FiO_2_ decrease (Figure 1(c)) and Evans blue dye accumulation (Figures 1(d) and 1(e)) in the mouse lung.

### 3.2. GSS Attenuates Sepsis-Induced Inflammatory Response in Mice

**Figure 2:**
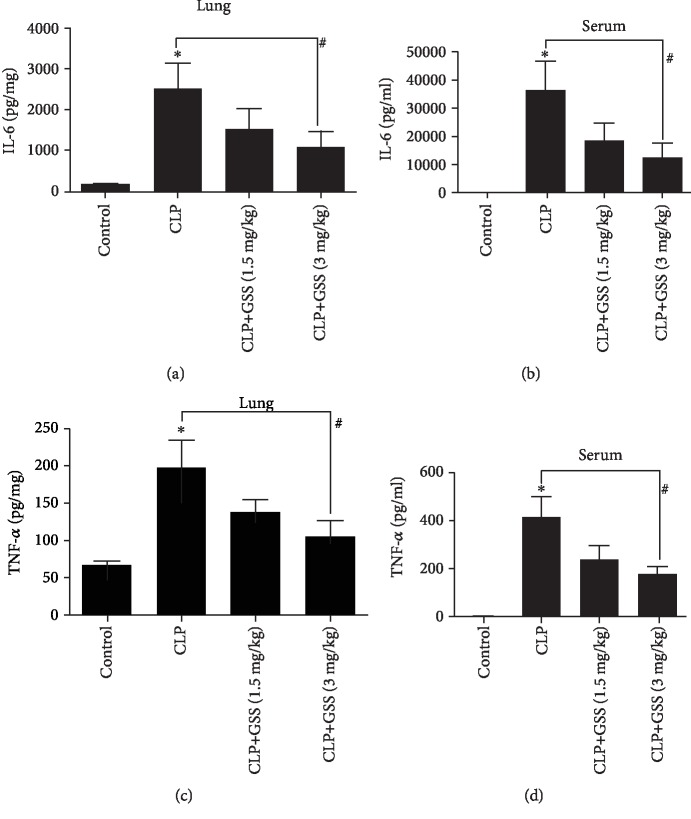
GSS blocks sepsis-induced inflammatory response in the lung tissue and the serum of mice. The mouse lungs harvested 24 h after CLP application. (a, c) Sepsis in C57BL/6J mice was induced by the CLP procedure after pretreatment of GSS, and the volume of TNF-*α* and IL-6 in the lung tissue was checked by ELISA. (b, d) The change of TNF-*α* and IL-6 in the serum of mice was detected by ELISA. ^∗^*P* < 0.05 versus negative control. ^#^*P* < 0.05 versus the corresponding CLP treatment group.

TNF-*α* and IL-6 are the important proinflammatory mediators, which are associated with systemic inflammatory response syndrome, acute lung injury, and mortality [15]. To further determine whether GSS inhibits sepsis-induced lung inflammation in vivo, we use ELISA analysis to check the changes of TNF-*α* and IL-6 in the lung tissue and serum, respectively. Compared with the control group, the CLP group obviously increased the expression of TNF-*α* and IL-6 in the lung tissue. However, these changes were significantly ameliorated by pretreatment with GSS (Figures 2(a) and 2(c)). At the same time, we also found that GSS effectively inhibited CLP-induced augment of TNF-*α* and IL-6 in the serum of mice (Figures 2(b) and 2(d)).

### 3.3. Effect of GSS on Cell Cytotoxicity in ECs

**Figure 3:**
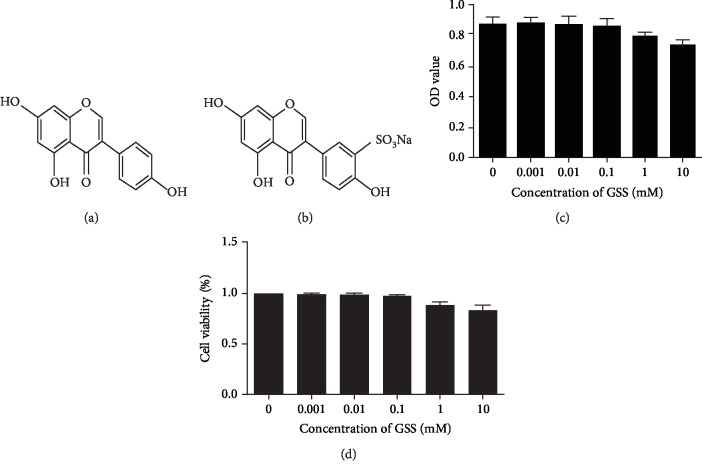
Chemical structure of GSS and its effect on EC viability. (a) The chemical structure of genistein. (b) The chemical structure of genistein-3′-sodium sulfonate. To observe the effect of GSS on the viability of ECs, cells were stimulated with 0, 0.001, 0.01, 0.1, 1, and 10 mM GSS for 24 h, respectively. The OD value (c) and EC viability (d) were measured by CCK-8 assay.

Based on CCK-8 cell viability assays, we checked the cytotoxicity of GSS in ECs. The chemical structure of genistein (Figure 3(a)) and GSS (Figure 3(b)). As shown in Figures 3(c) and 3(d), GSS has no obvious toxic effects on ECs at the concentrations below 0.1 mM. Moreover, though EC cell viability was reduced at concentrations of 1 and 10 mM, the results have no statistical significance compared with the control group. In this study, we further used GSS concentrations at 0.1 mM to explore its protective effect on LPS-induced EC injury.

### 3.4. GSS Inhibits LPS-Induced TNF-*α* and IL-6 Expression and Secretion in ECs

**Figure 4:**
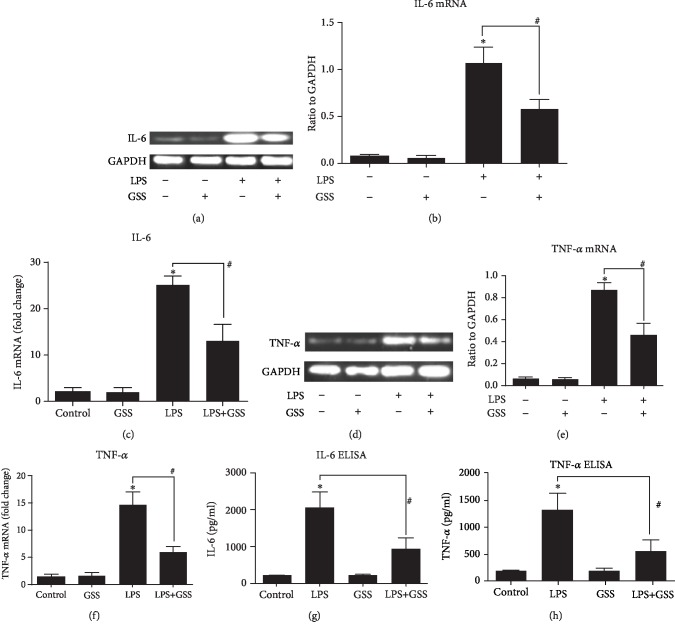
GSS inhibits LPS-induced TNF-*α* and IL-6 expression in ECs. After stimulation of LPS for 8 h, the endothelial cells were collected, and the mRNA and protein expressions of IL-6 and TNF-*α* were detected by RT-PCR and ELISA. (a) ECs were pretreated with GSS for 2 h and then exposed to LPS, and the expression of IL-6 was detected by RT-PCR. (b) The PCR result was presented as a histogram showing the band intensity values. (c) The RT-qPCR results of IL-6 were presented as a histogram. The relative amount was normalized to the housekeeping gene GAPDH. (d) ECs were pretreated with GSS for 2 h and then exposed to LPS, and the expression of TNF-*α* was detected by RT-PCR. (e) The PCR result was presented as a histogram showing the band intensity values. (f) The RT-qPCR results of TNF-*α* were presented as a histogram. The relative amount was normalized to the housekeeping gene GAPDH. (g) ECs were pretreated with GSS for 2 h and then stimulated with LPS; the level of IL-6 in supernatant of ECs was determined by ELISA analysis. (h) ECs were pretreated with GSS for 2 h and then stimulated with LPS; the level of TNF-*α* in the supernatant of ECs was determined by ELISA analysis. ^∗^*P* < 0.05 versus negative control. ^#^*P* < 0.05 versus the corresponding LPS treatment group.

To determine whether GSS inhibits sepsis-induced inflammatory response and ALI via lung vascular ECs, we further check the expression and secretion changes of TNF-*α* and IL-6 in ECs with and without LPS stimulation. After stimulation of LPS for 8 h, the endothelial cells were collected, and the mRNA and protein expression of IL-6 and TNF-*α* were detected by RT-PCR and ELISA. Compared with the control group, the LPS group obviously increased IL-6 and TNF-*α* expression in ECs. However, these changes were significantly inhibited by pretreatment with GSS (Figures 4(a)–4(f)). Similarly, we also found that GSS effectively reversed LPS-induced secretion of IL-6 and TNF-*α* in lung vascular ECs (Figures 4(g) and 4(h)).

### 3.5. LPS-Induced NF-*κ*B Activation Is Attenuated by GSS in ECs

**Figure 5:**
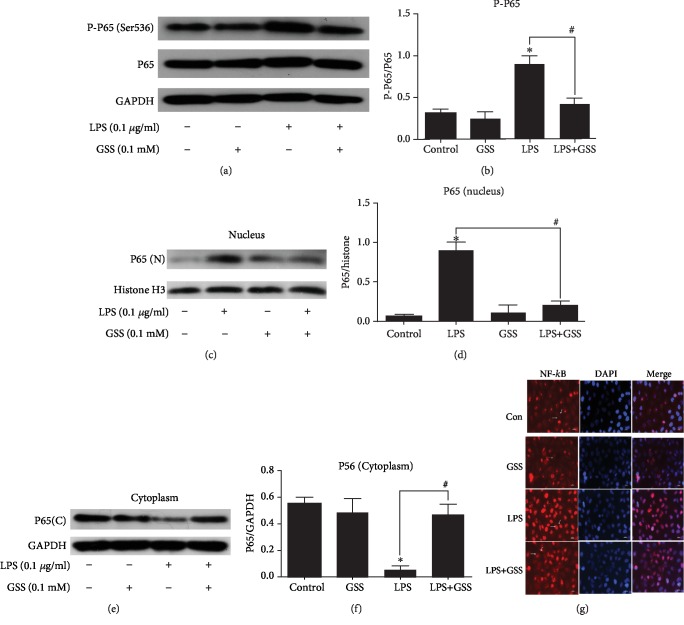
Effects of GSS on the LPS-induced activation of NF-*κ*B in ECs. (a, b) GSS had a significant inhibitory effect on LPS-induced increase of P-P65 expression compared to the LPS-stimulated group. (c, d) The expression of P65 in the nucleus of ECs was measured by Western blot analysis. (e, f) P65 expression in the cytoplasm of ECs was measured by WB assay. (g) The nuclear translocation of NF-*κ*B was analyzed using the confocal microscope (×200 magnification), which revealed that GSS effectively blocked LPS-induced P65 translocation into the nucleus of ECs. ^∗^*P* < 0.05 versus negative control. ^#^*P* < 0.05 versus the corresponding LPS treatment group.

NF-*κ*B is an important transcription factor and is implicated in the regulation of many inflammatory factor liberation, such as TNF-*α* and IL-6 [23]. In order to examine whether GSS inhibited LPS-induced TNF-*α* and IL-6 increase in ECs through NF-*κ*B signaling, we checked the expression and activation of NF-*κ*B in ECs. In this study, ECs were pretreated with GSS for 2 hours and then stimulated with LPS. Western blot analysis was conducted to examine the effects of GSS on the LPS-induced upregulation of NF-*κ*B in ECs. The results showed that GSS exhibited significant inhibitory effect on LPS-induced increase in NF-*κ*B expression (Figures 5(a) and 5(b)). NF-*κ*B is an important transcription factor, and the activation of NF-*κ*B will mediate its translocation from cytoplasm to nucleus. In order to examine the effect of GSS on LPS-induced NF-*κ*B activation, the nuclear and cytosolic extracts were isolated from ECs. The expression of NF-*κ*B in the cytosolic and nuclear fractions of ECs after treatment of LPS with/without GSS was checked using Western blot analysis. We found that LPS increased nuclear translocation of NF-*κ*B in ECs, but pretreatment of GSS prior to LPS stimulation significantly decrease the levels of NF-*κ*B in the nucleus of ECs (Figures 5(c)–5(f)). We further used fluorescence confocal microscopy to examine the subcellular localization and fluorescence intensity of NF-*κ*B. We found that NF-*κ*B was mainly localized in the nucleus of ECs in the LPS-stimulated group. However, preincubation with GSS effectively decreased LPS-induced NF-*κ*B translocation into the nucleus (Figure 5(g)).

### 3.6. GSS Regulates LPS-Induced Myd88 Expression in ECs

**Figure 6:**
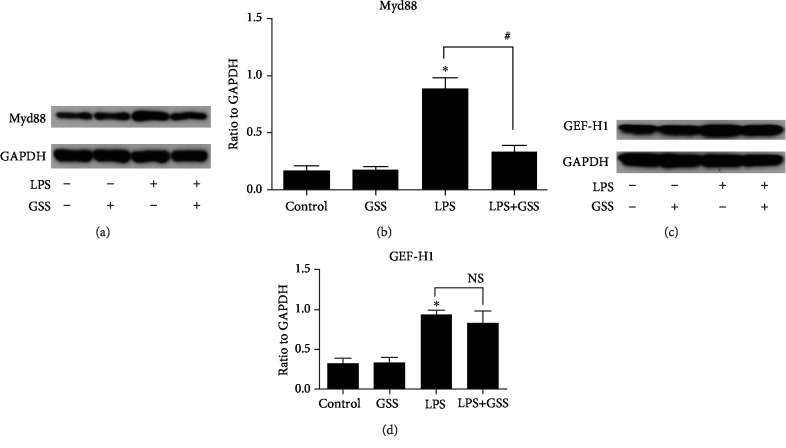
GSS block LPS-induced Myd88 expression in ECs. (a, b) ECs were incubated with GSS for 2 hours before LPS stimulation, and the expression of Myd88 was determined by Western blot. (c, d) ECs were pretreated with GSS for 2 hours followed by LPS stimulation, and the inhibitory effect on GEF-H1 was determined by Western blot. ^∗^*P* < 0.05 versus negative control. ^#^*P* < 0.05 versus the corresponding LPS treatment group.

NF-*κ*B activation is mediated by a wide variety of signals. Our previous studies found that both GEF-H1 and Myd88 are involved in the regulation of NF-*κ*B during LPS-induced EC injury [18]. In order to investigate the underlying mechanism of GSS on LPS-induced NF-*κ*B activation, we pretreated ECs with GSS and then added LPS. We performed a Western blot assay to verify whether GSS has the block effects on GEF-H1 and Myd88. We found that GSS effectively inhibited LPS-induced Myd88 expression (Figures 6(a) and 6(b)). However, GSS could not effectively block LPS-induced overexpression of GEF-H1 protein (Figures 6(c) and 6(d)). The above data indicated that GSS might protect against LPS-induced EC apoptosis via Myd88/NF-*κ*B signaling.

### 3.7. GSS Inhibits LPS-Induced TNF-*α* and IL-6 Expression in ECs via Myd88/NF-*κ*B Signaling Pathway

**Figure 7:**
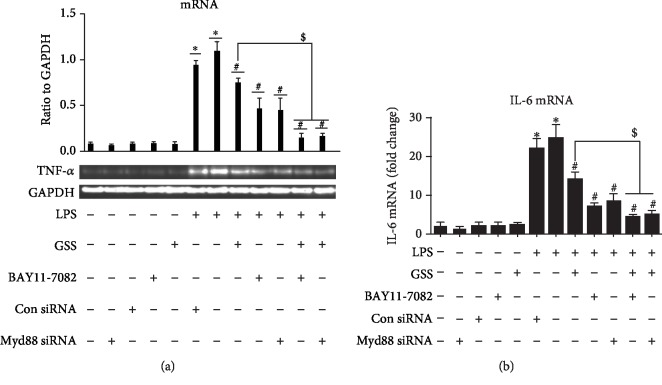
GSS inhibit LPS-induced IL-6 expression in ECs via Myd88/NF-*κ*B signaling pathway. (a) After transfection with Myd88 siRNA for 48 hours, ECs were treated with GSS for another 2 hours prior to the stimulation of LPS; the expression of IL-6 was determined by RT-PCR. Similarly, ECs were pretreated with BAY11-7082 (10 *μ*mol/l) and GSS for 2 hours and then incubated with LPS; the IL-6 expression was checked by RT-PCR. (b) The RT-qPCR results of IL-6 was presented as a histogram. The relative amount was normalized to the housekeeping gene GAPDH. ^∗^*P* < 0.05 versus negative control. ^#^*P* < 0.05 versus the corresponding LPS treatment group. ^$^*P* < 0.05 versus the corresponding GSS treatment group.

**Figure 8:**
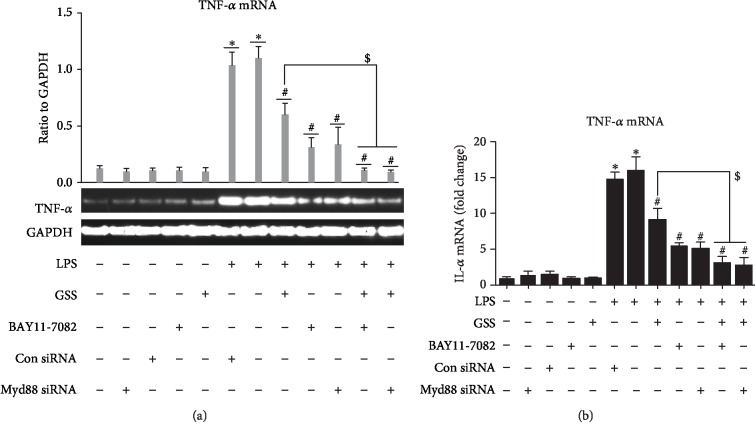
GSS inhibit LPS-induced TNF-*α* expression in ECs via Myd88/NF-*κ*B signaling pathway. (a) After transfection with Myd88 siRNA for 48 hours, ECs were treated with GSS for another 2 hours prior to the stimulation of LPS; the expression of TNF-*α* was determined by RT-PCR. Similarly, ECs were pretreated with BAY11-7082 (10 *μ*mol/l) and GSS for 2 hours and then incubated with LPS; the TNF-*α* expression was checked by RT-PCR. (b) The RT-qPCR results of TNF-*α* were presented as a histogram. The relative amount was normalized to the housekeeping gene GAPDH. ^∗^*P* < 0.05 versus negative control. ^#^*P* < 0.05 versus the corresponding LPS treatment group. ^$^*P* < 0.05 versus the corresponding GSS treatment group.

To further clarify the protective role of GSS on LPS-induced EC inflammatory response, we pretreated ECs with GSS, Myd88 siRNA, and BAY11-7082 (the specific inhibitor of NF-*κ*B) (10 *μ*mol/l) before the stimulation of LPS. As shown in Figures 7(a) and 7(b) and Figures 8(a) and 8(b), not only GSS but also inhibition of Myd88 or NF-*κ*B significantly reversed LPS-induced increase of TNF-*α* and IL-6. The above data indicates that GSS protects ECs against LPS-induced TNF-*α* and IL-6 augment via inactivation of Myd88/NF-*κ*B signaling pathway.

## 4. Discussion

ALI is a common complication of sepsis in which pulmonary endothelial cell dysfunction is regarded to be the primary initiator [24]. A large number of studies reported that the lung vascular endothelial cells are key modulators and orchestrators of ALI [25], and LPS cause lung endothelial cell dysfunction and ALI [26]. Lung endothelial cell injury and endothelial barrier failure are the key event in edema formation and inflammation in ALI [27].

ECs play a vital role in the progress of sepsis as first-line responders against invading pathogens [28]. When infectious bacteria release LPS into the bloodstream, LPS quickly identifies and binds receptors of the ECs, leading to a release of a series of cytokines and causing endothelial dysfunction [29]. Increased cytokines, such as TNF-*α* and IL-6, are hallmarks of many human inflammatory states, including sepsis [30, 31]. Previous data have suggested protective effects of genistein such as antitumor, antioxidative, and antiapoptotic functions [32–34]; however, the anti-inflammatory effect of GSS, a new compound derived from genistein, is still incompletely understood. In the present study, we demonstrated that the GSS was able to inhibit LPS-induced lung microvascular EC injury and sepsis-induced ALI. The underlying mechanisms involved in the above process were associated with reducing LPS-induced overexpression of TNF-*α* and IL-6 in the lung microvascular ECs by GSS, which was mediated by the downregulation of the Myd88/NF-*κ*B signaling pathway.

In septic conditions, the lung is a frequently affected organ. Infection-induced lung pathological changes and subsequent ALI are the major cause of death in septic patients [7, 35, 36]. Multiple previous studies have revealed the mechanisms involved in LPS-induced ALI [20, 37, 38]; however, synthetic chemical inhibitors of pathogenesis had more side effects, limiting their widespread clinical use. The natural flavones that occurred in high concentrations in fruits gradually attracted people's attention. Some previous studies have focused on the effects of genistein on brain injury and suggested that genistein protected the brain against inflammation. For example, Mirahmadi et al. reported that genistein alleviated neural inflammation and cognitive dysfunctions [39]. Jeong et al. demonstrated that genistein inhibited activation of microglial cells and release of inflammatory cytokines [19]. Other scholars recently revealed that GSS could protect cortical neurons from focal cerebral ischemia-induced apoptosis of neurocytes [5]. Therefore, it was of interest to understand the effects of GSS on LPS-induced ALI. The lesions in LPS-induced ALI primarily include pulmonary edema and infiltration of multiple inflammatory cells [37]. In the present study, first, we evaluated the role of GSS in change of pulmonary histopathology, oxygen index, and ratios of wet/dry. Our results show that GSS can effectively reverse the LPS-induced decrease of the oxygen index, increase of wet/dry ratios, and lung pathological changes. These results indicated that GSS plays a substantial protective role in LPS-induced lung injury and mouse mortality. To further characterize the effect of GSS on LPS-induced lung overinflammation, we analyzed the expression of TNF-*α* and IL-6 in pulmonary tissue and serum of mice by ELISA. We found that GSS significantly inhibited LPS-induced overexpression of TNF-*α* and IL-6. Together with previous reports [5], these results suggest that GSS not only plays a protective role in the brain injury but also effectively inhibits LPS-induced lung injury via suppression of inflammation.

To elucidate the mechanism by which GSS exerts protective effect on lung microvascular ECs to provide cell-based evidence for the therapeutic effect of GSS in LPS-induced ALI, we further tested the role of GSS on the expression and release of TNF-*α* and IL-6 in ECs in vitro. Consistent with our above findings in vivo, we also observed that GSS significantly inhibited the expression of TNF-*α* and IL-6 in ECs. Analogous to previous reports [23], these data provide further evidence that the LPS-induced EC injury and release of TNF-*α* and IL-6 are the leading reason of LPS-induced ALI.

NF-*κ*B is a pleiotropic transcription factor that plays an important role in activation of proinflammatory genes and is believed to be a promising target for the treatment of inflammation [40]. In general, the inactive form of NF-*κ*B is present in the cytoplasm. When it is activated by phosphorylation, NF-*κ*B translocates into the nucleus, where active NF-*κ*B binds to specific DNA sequences, thereby increasing the expression of many downstream genes, including TNF-*α* and IL-6 [41]. In this study, GSS exhibited statistically significant inhibition of the activation of NF-*κ*B and attenuated LPS-induced NF-*κ*B translocation from the cytoplasm to the nucleus in ECs. Our data suggested that NF-*κ*B signaling might be implicated in GSS-induced inhibition of TNF-*α* and IL-6 in LPS-stimulated ECs.

It is well known that there are many upstream proteins regulating the activity of NF-*κ*B [42]. Among these signaling pathways, Myd88-dependent and GEF-H1-dependent manner-mediated regulation of NF-*κ*B is the main pathways implicated in LPS-induced endothelium injury [18]. To gain further insight into the protective role of the GSS on LPS-induced overexpression of TNF-*α* and IL-6 and to understand the intrinsic molecular mechanism, we assessed the expression and activity changes of Myd88 and GEF-H1 after pretreatment with GSS in LPS-incubated ECs. In the present study, we found that GSS protected against LPS-induced overexpression of proinflammatory mediators in endothelial cells via Myd88 signaling pathway. Furthermore, combined application of GSS and Myd88 siRNA and also combined application of GSS and NF-*κ*B inhibitor produce an increased inhibitory effect on LPS-induced expression of TNF-*α* and IL-6 compared with GSS alone. These results indicate that GSS partly block proinflammation cytokine-associated EC injury through the Myd88/NF-*κ*B/TNF-*α*/IL-6 signaling pathway in vitro.

**Figure 9:**
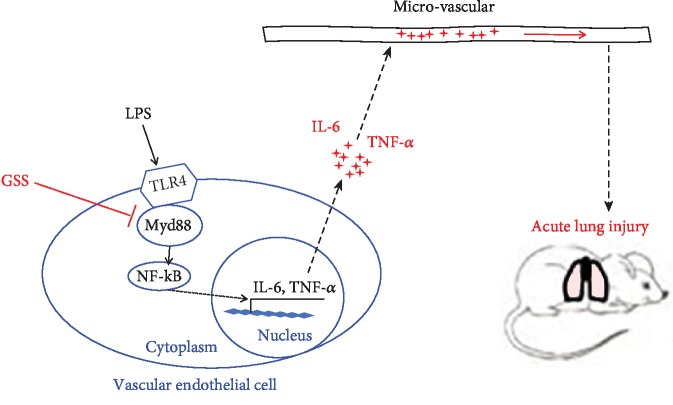
Schematic figure of possible signaling mechanisms of GSS in the inhibition of LPS-induced lung vascular endothelial cell inflammatory response and subsequent ALI in mice.

As shown in Figure 9, we explored for the first time the role of GSS on the anti-inflammatory properties of lung microvascular endothelial cells in this study. We also provide new evidence that the molecular mechanism of the protective effect of GSS on the production of LPS-induced TNF-*α* and IL-6 is mediated in a Myd88/NF-*κ*B-dependent manner. However, accompanied by the injury of lung epithelial cells and vascular endothelial cells, sepsis-induced acute lung injury began to develop [43]. In addition to LPS-induced overexpression of inflammatory mediators in endothelial cell, LPS-induced inflammation activation in epithelial cells also plays an important role in the process of sepsis-induced lung injury [44]. Hence, GSS might also block LPS-induced release of inflammatory mediators in lung epithelial cells, which need to be studied further.

## 5. Conclusion

In summary, the present study indicated that GSS has the inhibitory effect on LPS-induced acute lung injury and pulmonary vascular EC inflammation response. GSS significantly attenuated lung histopathological changes by reducing lung microvascular permeability and inflammatory factor release. We further revealed that the Myd88/NF-*κ*B/TNF-*α*/IL-6 signaling pathway was the important target of GSS. All above results suggest that GSS may be a novel therapeutic agent for the prevention of EC inflammation-associated lung injury during septic condition.

## Data Availability

The data used to support the findings of this study are included within the article at the last part of this manuscript.
